# Priming with copper-chitosan nanoparticles elicit tolerance against PEG-induced hyperosmotic stress and salinity in wheat

**DOI:** 10.1186/s13065-022-00813-1

**Published:** 2022-04-01

**Authors:** Tahir Farooq, Zaib Un Nisa, Amjad Hameed, Toheed Ahmed, Arruje Hameed

**Affiliations:** 1 Department of Applied Chemistry, Government College University, Faisalabad, Pakistan; 2 Department of Biochemistry, Government College University, Faisalabad, Pakistan; 3 Nuclear Institute for Agriculture and Biology (NIAB), Jhang Road, Faisalabad, Pakistan; 4 Department of Chemistry, Riphah international university, Faisalabad, 380000 Pakistan

**Keywords:** Wheat priming, Cu-chitosan nanoparticles, PEG-induced hyperosmotic stress, Salinity, Stress tolerance

## Abstract

In this study Cu-chitosan nanoparticles (Cu-CNP) have been employed as eco-friendly and safer priming agents to induce salt and PEG-induced hyperosmotic stress tolerance in wheat seedlings. Seed priming is a facile on-farm stress management technique that requires a little amount of priming agent and minimizes the eco-toxicological effects on soil fertility. The wheat seeds were primed with 0.12% and 0.16% Cu-CNP for eight hours and were allowed to germinate under normal, PEG-induced hyperosmotic stress (15% PEG-6000  – 3.0 Mpa) and salt stress (150 mM). For comparison, non-primed and hydro-primed seeds were also allowed to germinate as control under the same conditions. The biochemical analyses suggested the priming treatments enhanced the POD activity under salt stress but it was decreased under PEG-induced hyperosmotic stress. Priming with 0.12% Cu-CNP induced a significant increase in CAT while the opposite effect was observed in 0.16% treated seedling under stress and non-stress conditions. Both priming treatments did not allow the over-expression of SOD under both stress conditions. The total phenolic contents were also decreased significantly under all conditions. Except for priming with 0.16% Cu-CNP under PEG-induced hyperosmotic stress, a suppression in MDA was observed under both stress conditions. Surprisingly, the Cu-CNP priming induced a significant increase in β-carotenoids, total carotenoids, chlorophyll a, b and total chlorophyll under normal and stress conditions. In conclusion, the controlled expression of enzymatic antioxidants, low contents of non-enzymatic antioxidants and suppression of MDA mirror the stress mitigating role of Cu-CNP against PEG-induced hyperosmotic stress and salinity. The stress-insulating potential has also been reinforced by the enhanced production of plant and photosynthetic pigments. All these priming-induced biochemical changes produced positive effects on growth and germinating parameters in wheat seedlings under PEG-induced hyperosmotic stress as well as salinity.

## Introduction

The germination, growth and development of cereal crops have greatly been affected by the ecotoxicological conditions and environmental stresses over the last few decades. The water resource deficits due to the rising global warming result in severe water shortages around the globe. Water deficiency poses serious threats to crop production as drought stress in various parts of the world especially in developing countries [1]. Drought induces a variety of changes at sub-cellular levels including metabolic processes, biochemical and physiological attributes, and morphological characteristics. Such undesirable changes in growth promontory parameters inhibit the growth and limit the production of crop plants [2].

Salinity has emerged as the most brutal environmental contamination and devastating abiotic stress which adversely affects the enzymatic activities, nutrient uptake, photosynthesis and water uptake in plants. The nutritional imbalance and damaged ultracellular components disrupt the normal metabolic processes consequently reduce germination percentage, development and yield of crops [3].

Among the cereal crops, wheat is a major staple food and a source of about 55% of carbohydrates requirements around the globe. Various biotic and abiotic stresses including drought and salinity severely affect wheat production, especially in developing countries. The wheat seed germination and seedling growth do experience the above said negative impacts on metabolic processes, biochemical and physiological attributes under drought as well as salt stress. Consequently, they cause serious depression in germination percentage, biomass production and grain yield [4, 5].

Over the years, various stress management strategies have been evolved to induce tolerance against drought and salt stress for the productivity of wheat. In this regard, different approaches including screening of better performing genotypes, the introduction of tolerance-inducing genes and remodeling of conventional breeding methods have met limited success because they are time-consuming and lose efficiency over time. In recent years, the use of exogenous phytoprotectants as growth promoters and stress emulators have received considerable attraction due to the ease of on-farm handling and applications as priming agents, foliar spray and in soil drenching [6, 7]. However, the perpetual applications of phytoprotectants especially synthetic agrochemicals have raised several unavoidable environmental issues. The rampant and uncontrolled use of synthetic chemicals pose serious ecotoxicological threats and they destroy much-needed soil microbial community due to off-target interactions. They also produce resistance in plant pathogenic microbes thus lead to qualitative and quantitative loss of agricultural productivity [8]. Priming has emerged as an alternative approach in which seeds are pre-treated with a minimum amount of agrochemicals to avoid undesirable soil contamination through drenching and foliar sprays. This facile pre-treatment technique enhances the quality of seeds for fast germination, stress tolerance and high productivity of crops [9]. In fact, it triggers specific metabolic changes, antioxidant enzymes and protein synthesis ensuring better crop performance under various biotic and abiotic stresses [10, 11].

In the last few years, attention have been diverted to the development of effective and safer biomaterial-based biodegradable agrochemicals as a counteracting strategy for environmental safety and pollution-related challenging issues. In this regard, chitosan has become a promising biopolymer due to its biodegradable, biocompatible and nontoxic nature [12]. It has established its worth as a plant growth-promoting agents, immunomodulator and non-hazardous stress emulator with wide applications in agriculture for the enhancement of crop productivity. It has widely been exploited as a growth stimulator, antioxidant defense booster, stress emulator and yield promoter in wheat under a variety of stress conditions including drought and salinity [13].

Nanotechnology has been revolutionizing almost all aspects of human life including the establishment of a very vital role in agriculture [14, 15]. The nanomaterials like nanoparticles have gained a crucial role in defense system activation, growth enhancement, disease and pest management strategies in plants [16]. Although, several studies have raised concerns regarding the negative impacts of nanoparticles on the environment and human health over the last few years [17]. However, the chitosan nanoparticles (CNPs) have shown significantly low toxicity and their safety profiles suggested them safer even for gene delivery to the spinal cord [18]. In recent years, CNPs have been evaluated for their potential to enhance seed germination, seedling growth and defense responses in plants. The foliar applications of CNPs promoted growth and yield of coffee [19], French beans [20] and wheat [21]. The foliar applications have also been used to induce salt stress tolerance in beans [22]. Mechanistically, chitosan is known to boost the immune system by inducing the synthesis of glucanase and chitinase enzymes. Further, the chitosan applications enhance the production of nitric oxide which is a vital signaling molecule modulating the defense responses in plants. The CNPs induce the synthesis of plant defense-related secondary metabolites including phytoalexins, flavonoids and lignin. The production of these sec-metabolites is mediated by up-regulating the activity of Tyrosine Ammonia-Lyase (TAL) on the phenylpropanoid synthesis pathway [13, 23].

Cu is a key structural component of many enzymes crucial for cellular redox and electron transfer reactions in plants, thus an important micronutrient with the capacity to boost growth and developmental phases. Reckoning this important fact, Cu-CNPs have been developed recently and evaluated for their potential to boost growth, enhance development and disease management in plants [24, 25]. The sustained release of Cu from Cu-CNPs makes it a far superior bioactive system compared to simple CNPs [26].

Considering the high bioactive potential of Cu-CNPs and the ease of on-farm priming applications, we used Cu-CNPs as priming agents to induce PEG-induced hyperosmotic stress and salt tolerance in wheat seedlings.

## Materials and methods

### Preparation and characterization of Cu-CNPs

**Fig. 1:**
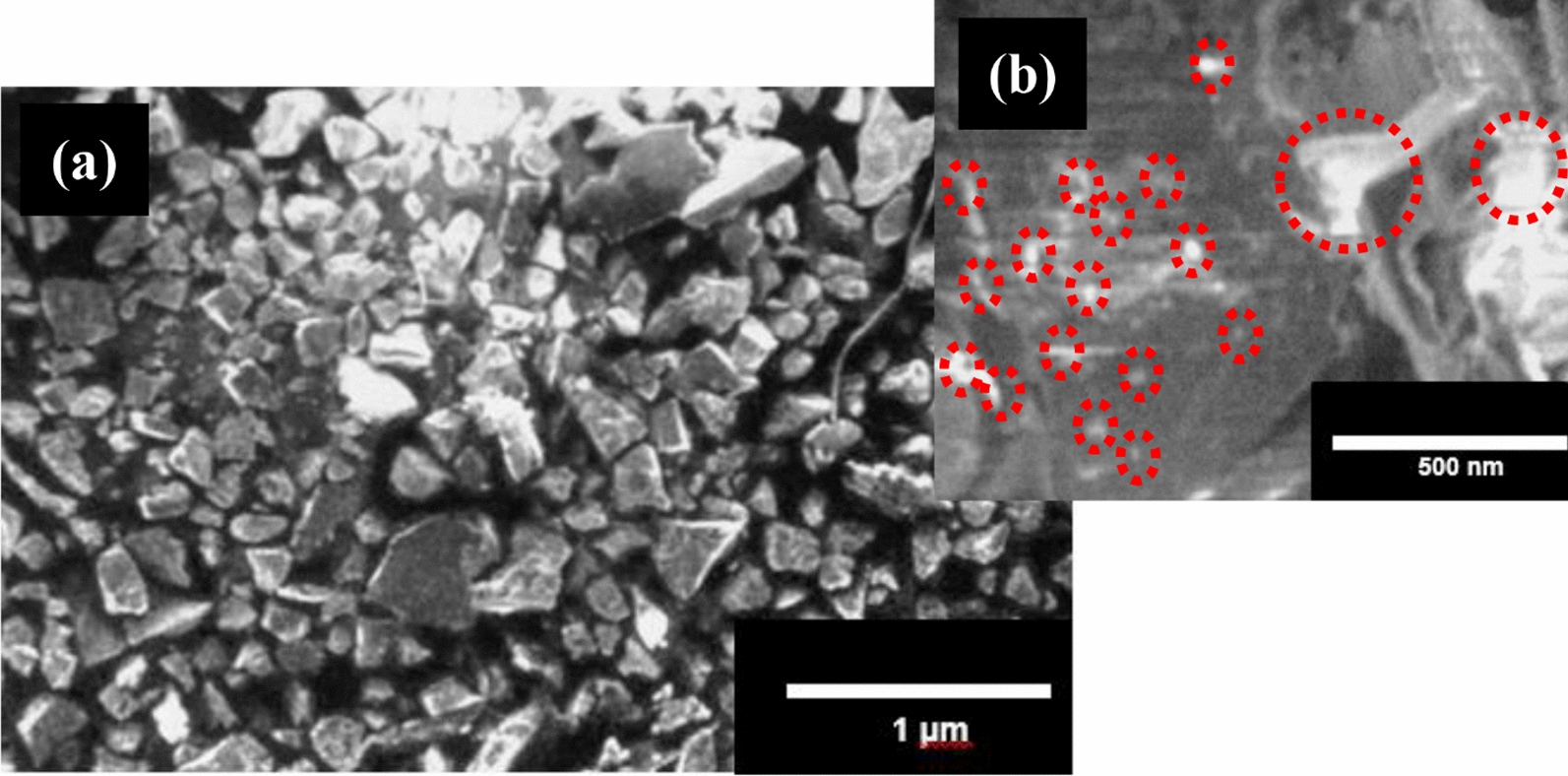
SEM of the prepared Cu-CNPs

**Fig. 2:**
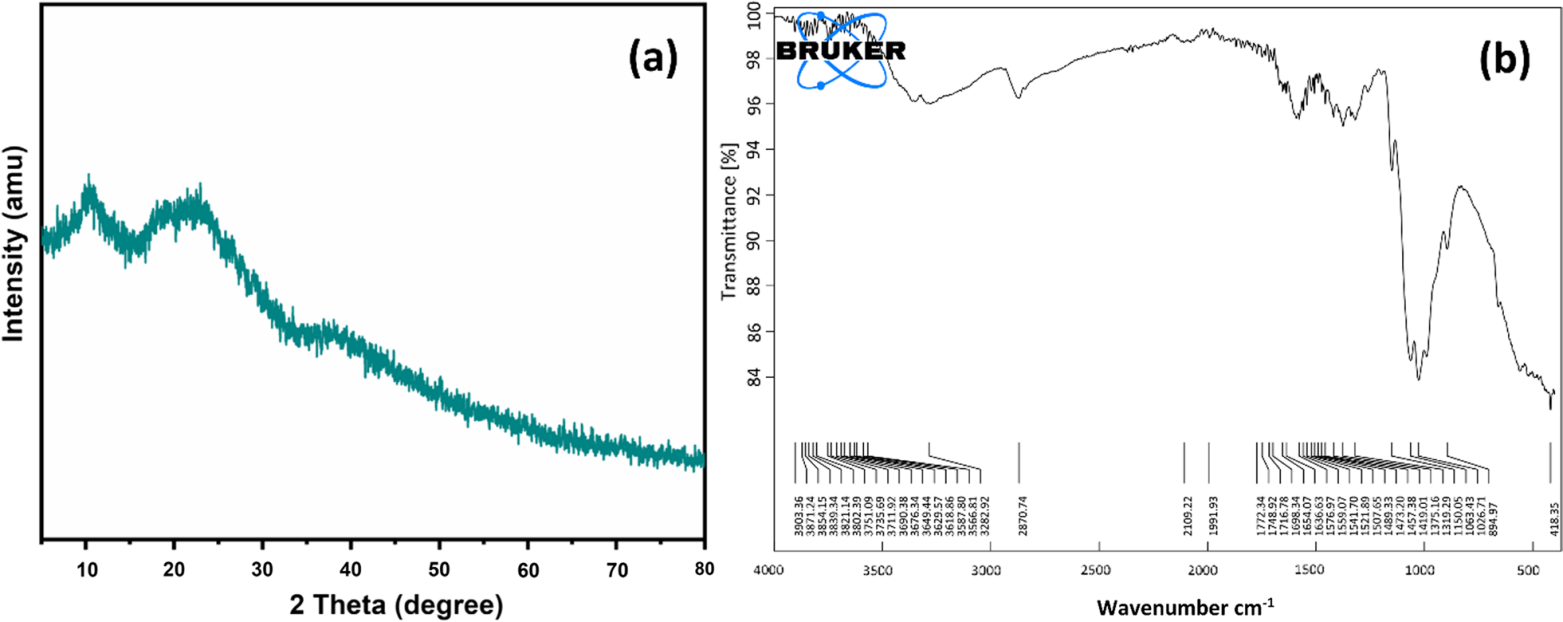
Characterization of prepared Cu-CNPs

The Cu-CNPs were prepared by following reported methods [25, 27] and subsequently characterized by SEM, XRD and FTIR Figs. (1 and 2). The Fig. 1 (a) shows the morphology of synthesized Cu-CNPs. The prepared Cu-CNPs showed cubical structure with varying size (b) shows that Cu NPs embedded on the surface of chitosan of irregular shape with the cubical aggregated structure of the prepared Cu-CNPs. The red circular lines indicate the NPs from which the approximately spherical shape of the poly-dispersed Cu NPs can be seen. The particles were found to be in the range of 19–21 nm in size as estimated using the Debye–Scherrer equation with data obtained from XRD.

The Fig. 2a showed the XRD diffraction pattern of the prepared Cu-CNPs. XRD diffraction peaks at 2 theta of 12 ^°^ and 20 ° correspond to (020), (220), which exhibited that the Cu NPs had been coated on chitosan. The XRD spectrum also shows the peaks in the range of 22.8 °, 25.4 °, 32.1 °, 45.4 °, 53.1 °, 56.1 °, 65.8 °, 69.2 °, and 77.3 °, which correspond to the (021), (021), (110), (111), (020), (202), ( − 113), (202), and (111) planes, respectively [28]. The XRD spectrum showed that the prepared Cu-CNPs had the cubic-crystalline structure, and the corresponding planes were matched with the JCPDS file number (JCPDS NO: 05-0061) [29]. The average crystalline size was calculated by using simple Debye–Schere’s formula:

$$\text{D}=0.9 \uplambda+\upbeta cos$$

whereas D corresponds to the average size of the nanoparticles, λ is the wavelength of X-ray, θ is the diffraction angle and β is the FWHM (full width at half maximum) in radians a [30]. The average mean crystallite size was found to be 19 nm from all breath of the refraction. The Fig. 2b show FTIR of the prepared Cu-CNPs which was found in agreement with previously reported literature values.

### Seed priming and germination studies

Wheat seeds (*Triticum aestivum* L. cv. AARI-2011) were received from Ayub Agriculture Research Institute, Faisalabad, Pakistan and primed by treating with 0.12% and 0.16% Cu-CNP solutions (prepared by sonication for 30 min) for 8 h. After that, they were washed with distilled water and re-dried under the shade at 26 ± 2 °C. Some seeds were also hydro-primed by soaking in distilled water for the same length of time. The Cu-CNP primed, hydro-primed and non-primed control seeds were allowed to germinate in separate petri dishes under normal, PEG-induced hyperosmotic stress (15% PEG-6000) (− 3.0 Mpa) [31] and salt stress (150 mM) conditions [32].

### Germination test and seedling vigor

The International Rules for Seed Testing (ISTA, 1985) were followed to estimate the termination potential of the primed and non-primed wheat seeds. At 25 °C four replicates of 25 seeds were germinated in 12 cm diameter petri dishes for the estimation of seed germination and seedling vigor under normal conditions in the growth chamber. The osmotic stress conditions were imposed by using 5 ml of 15% polyethylene glycol 6000 solution (− 3.0 Mpa) instead of water in each petri dish. Counts of germinating seeds were made every 6 h, starting on the first day of imbibition, and terminated when maximum germination was achieved. A seed was recorded as germinated when coleoptile and radicle lengths reached 2–3 mm.

Mean germination time (MGT) was calculated according to the equation of Ellis and Roberts (1981) and expressed as

$${\text{MGT (h)}} =\sum {{\text{hn}}} /\sum {\text{n}}$$

where h is the number of hours from the beginning of the germination test and n is number of seeds germinating on hours h.

### Growth response

After collecting the data for germination, seedlings from the above said experiments for seed germination tests were allowed to grow for growth response and biochemical studies. 7-day old seedlings were then harvested for comparison of growth and biochemical parameters under normal, salt and PEG-induced hyperosmotic stress after seed priming treatments. The fresh weight of seedlings was recorded immediately after harvesting to avoid any evaporation. For dry weight estimations, a set of harvested seedlings were kept at 90 °C till drying. Seedling, root and shoot dry weights were measured after complete drying when there was no further decrease in weight. Root and shoot lengths were measured by spreading them on a scale calibrated in cm.

### Antioxidant analyses

Known procedures were followed to analyze the contents of biomolecules (proteins and total sugars) [33] and hydrolytic enzymes (α- amylase and protease) [34, 35]. The enzymatic antioxidant (POD, CAT and SOD), [36, 37] non-enzymatic antioxidants (TPC) [38] and stress biomarker (MDA) [39] were measured by following the well-established methods. Further, the known assays were also adapted to measure the plant (lycopene, β-carotenoids, total carotenoids) [40] and photosynthetic pigments (chlorophyll a, b and total chlorophyll) [41] in wheat seedlings. Further, the germination and growth parameters were also studied for established seedlings.

All these afore-said biochemical analyses and germination studies were performed using wheat seedlings originated from hydro-primed and Cu-CNP primed seeds grown under normal, PEG-induced hyperosmotic stress and salt stress conditions and were compared with non-primed controls under the same conditions.

Significance of the recorded data was tested using analysis of variance and Tukey’s (HSD) test at value p < 0.05 and where applicable at p < 0.01 using XL-STAT software. The values are presented in graphs as mean ± SD.

## Results

### Effect of Cu-CNPs priming on germination parameters

**Fig. 3:**
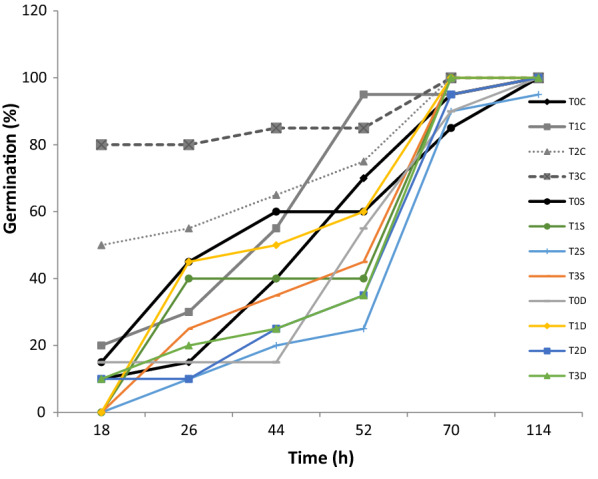
Effect of Cu-CNPs priming on germination% under control, salt and PEG-induced hyperosmotic stress. Non-primed (T0C), Hydro primed (T1C), 0.12% Cu chitosan NPs primed (T2C), and 0.16% Cu chitosan NPs primed (T3C) wheat seeds under control condition; Non-primed (T0S), Hydro primed (T1S), 0.12% Cu chitosan NPs primed (T2S), and 0.16% Cu chitosan NPs primed (T3S) wheat seeds under salt stress; Non-primed (T0D), Hydro primed (T1D), 0.12% Cu chitosan NPs primed (T2D), and 0.16% Cu chitosan NPs primed (T3D) wheat seeds under PEG-induced hyperosmotic stress

Seed primed with 0.16% Cu-CNPs under normal conditions showed early germination and the best germination rate compared to all other seeds under control and stress conditions. As expected, both salt and PEG-induced hyperosmotic stress reduced the germination rates of the untreated seeds and the latter induced a more noticeable negative effect. However, priming treatments with Cu-CNPs helped to reduce the negative impacts of both stresses on germination rate (Fig. 3).

No significant difference in germination percentage of seeds was observed under normal, salt and PEG-induced hyperosmotic stress conditions (Table 1). Wheat seeds primed with 0.12% Cu-CNPs showed maximum mean germination % under salt and PEG-induced hyperosmotic stress conditions. In general, there was no significant difference in the mean germination % of seeds under normal, salt and PEG-induced hyperosmotic stress conditions. Under normal conditions, the priming treatments enhanced the germination index. However, the germination index decreased under stress conditions in treated and non-treated seeds. The Cu-CNP priming treatments enhanced the vigor index only under normal conditions. Both PEG-induced hyperosmotic stress and salt stress reduced the vigor index and the later induced maximum reduction in primed and non-primed seeds. Both stresses reduced the germination energy in primed and non-primed germinating wheat seeds. However, hydro-priming induced a significant increase in germination energy compared to control under normal conditions.

**Table 1:** Effect of Cu- CNP priming treatments on germination and growth parameters

Germination /growth parameters	Treatments											
	Non- Stress				Under PEG-induced hyperosmotic stress				Under salt stress			
	Control	Hydro-priming	0.12% Cu- CNP	0.16% Cu- CNP	Control	Hydro-priming	0.12% Cu- CNP	0.16% Cu- CNP	Control	Hydro-priming	0.12% Cu- CNP	0.16% Cu- CNP
Final Germination percentage (%)	100% a	100% a	100% a	100% a	100% a	100% a	100% a	100% a	100% a	100% a	95% a	100% a
Mean Germination Time (MGT) (hours)	72.92 ab	65.95 abc	61.77 bc	56.76 c	75.76 ab	70.35 abc	77.9 a	75.96 ab	68.12 abc	73.03 ab	81.5 a	75.26 ab
Germination index (GI) %	8.4 bc	11.9 bc	15.46 ab	20.63 a	7.59 bc	9.08 bc	6.77 c	7.37 bc	10.62 bc	8.0 bc	5.17 c	7.21 c
Vigor index (VI) %	0.535 c	0.582 b	0.762 a	0.779 a	0.533 c	0.243 e	0.508 cd	0.499 d	0.194 f	0.153 g	0.237 e	0.199 f
Germination Energy (GE)	34.0 abc	45.5 a	16.5 bcd	8.5 d	41.05 ab	15.0 cd	12.5 cd	12.5 cd	6.0 d	4.0 d	7.0 d	13.5 cd
Shoot length (Centimeters)	5.400 b	5.835 b	7.640 a	7.805 a	5.330 b	2.430 c	5.160 b	4.990 b	1.945 c	1.530 c	2.500 c	1.985 c
Root length (Centimeters)	4.13 b	3.90 b	6.81 a	8.12 a	8.46 a	3.56 b	7.68 a	7.91 a	2.05 c	1.95 c	3.32 bc	3.80 b

Mean values of four replicates presented. Within a row, means sharing the same letters are non-significantly different (*p* > 0.05 and *p* > 0.01) according to the Tukey’s Test (HSD)

### Effect on growth parameters

Wheat seedlings originated from Cu-CNPs treated seeds showed a significant increase in root length under normal conditions and salt stress compared to controls (Table 1). However, the Cu-CNP priming treatments did not produce a significant difference in root length compared to non-primed under PEG-induced hyperosmotic stress. A significant increase in shoot length was noted in seedlings originated from Cu-CNP treated seeds under normal conditions. However, these priming treatments did not produce any significant difference in shoot length both under salt and PEG-induced hyperosmotic stress compared to control.

### Effect on protein and sugar contents

Both priming treatments increased proteins under PEG-induced hyperosmotic stress whereas treatment with 0.16% Cu-CNPs enhanced the protein contents under normal conditions. The salt stress reduced the protein contents in seedlings originated from primed seeds compared to control (Table 2). Priming with 0.12% Cu-CNPs induced a significant increase in total sugars under normal and salt stress conditions. While no other treatment produced any significant change in total sugars under any condition (Table 2).

**Table 2:** Effect of Cu- Chitosan nanoparticle priming treatments on biomolecules and hydrolytic enzymes

Biomolecules/ hydrolytic enzymes	Treatments											
	Non-Stress				Under PEG-induced hyperosmotic stress				Under salt stress			
	Control	Hydro-priming	0.12% Cu- CNP	0.16% Cu- CNP	Control	Hydro-priming	0.12% Cu- CNP	0.16% Cu- CNP	Control	Hydro-priming	0.12% Cu- CNP	0.16% Cu- CNP
Protein (mg/gf.wt)	151 f	287 b	140 f	267 c	184 e	150 f	287 b	303 b	376 a	215 d	174 e	304 b
Total Sugars (mg/g. f.wt)	91 cd	82 de	134 a	79 e	90 cd	80 e	93 c	91 cd	79 e	81 e	106 b	85 cde
α-Amylase (mg/g F.wt.)	112 b	123 b	41 e	73 d	123 b	72 d	14 f	71 d	193 a	73 d	54 e	91 c
Protease (units/g F.wt.)	8706 b	8629 b	7440 de	8197 bc	7904 cd	7050 ef	7491 de	9747 a	7440 de	10,150 a	6491 f	10,277 a

Mean values of three replicates presented. Within a column, means sharing the same letters are non-significantly different (*p* > 0.05 and *p* > 0.01) according to the Tukey’s Test (HSD)

### Effect on hydrolytic enzymes

The Cu-CNPs priming significantly reduced the α-amylase activity under normal and stress conditions (Table 2). While the maximum negative impact was recorded in seedlings originated from seeds primed with 0.12% Cu-CNPs. The priming treatments decreased the protease activity under normal conditions. Both priming treatments displayed opposite effects on protease activity whereas the maximum enhancement was induced by 0.16% Cu-CNP treatment compared to control under both stresses.

### Effect on enzymatic and non-enzymatic antioxidants

The Cu-CNPs priming reduced the POD activity significantly compared to control under normal conditions (Table 3). Under salt stress, the priming with 0.12% Cu-CNPs enhanced the POD activity while 0.16% reduced its activity significantly compared to control under salt stress. Both priming treatments induced a significant reduction in POD activity under PEG-induced hyperosmotic stress while the most pronounced impact was observed with 0.12% Cu-CNP priming. Under normal conditions, the treatment with 0.16% Cu-CNP enhanced the SOD activity significantly compared to control. Due to priming treatments, the SOD activity decreased both under PEG-induced hyperosmotic stress and salinity however, this reduction was less significant under salt stress. The maximum CAT activity was observed as a result of 0.12% Cu-CNPs priming under normal, salt and PEG-induced hyperosmotic stress conditions. However, the priming with 0.16% Cu-CNPs reduced the CAT activity under all conditions. The priming treatments reduced the TPC under all conditions however, the decrease was non-significant under PEG-induced hyperosmotic stress.

**Table 3:** Effect of Cu- CNP priming treatments on enzymatic and non- enzymatic antioxidants

Enzymatic /Non-Enzymatic Antioxidants	Treatments											
	Non- Stress				Under PEG-induced hyperosmotic stress				Under salt stress			
	Control	Hydro-priming	0.12% Cu- CNP	0.16% Cu- CNP	Control	Hydro-priming	0.12% Cu- CNP	0.16% Cu- CNP	Control	Hydro-priming	0.12% Cu- CNP	0.16% Cu- CNP
Catalase (CAT) (units/g f.wt)	437 cd	298 e	665 a	277 e	435 cd	470 c	652 a	445 cd	535 b	432 cd	650 a	417 d
Peroxidase (POD) (units/g f.wt)	29,252 a	6392 f	2913 g	6193 f	14,786 c	4139 g	3947 g	7470 f	9662 e	23,258 b	12,167 d	23,122 b
Superoxide dismutase (SOD) (units/g f.wt)	113 g	123 fg	121 fg	139 de	171 a	164 ab	148 cd	152 bcd	147 cd	142 de	160 abc	132 ef
Total Phenolics (TPC) (µM/g f.wt)	37,220 a	28,377 c	29,710 bc	27,750 c	21,822 de	21,053 e	24,277 d	18,150 f	32,322 b	19,400 ef	24,452 d	19,385 ef

Mean values of four replicates presented. Within a row, means sharing the same letters are non-significantly different (*p* > 0.05 and *p* > 0.01) according to the Tukey’s Test (HSD)

### Effect on lipid peroxidation and pigments

**Fig. 4:**
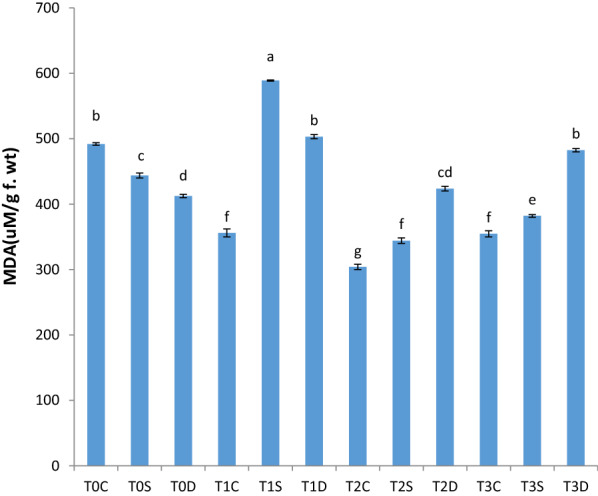
Effect of Cu-CNPs priming on MDA content under control, drought, and salt stress. Non-primed (T0C), Hydro primed (T1C), 0.12% Cu chitosan NPs primed (T2C), and 0.16% Cu chitosan NPs primed (T3C) wheat seeds under control condition; Non-primed (T0S), Hydro primed (T1S), 0.12% Cu chitosan NPs primed (T2S), and 0.16% Cu chitosan NPs primed (T3S) wheat seeds under salt stress; Non-primed (T0D), Hydro primed (T1D), 0.12% Cu chitosan NPs primed (T2D), and 0.16% Cu chitosan NPs primed (T3D) wheat seeds under PEG-induced hyperosmotic stress

Priming with Cu-CNPs reduced the MDA contents under normal, salt and PEG-induced hyperosmotic stress except 0.16% treatment under PEG-induced hyperosmotic stress (Fig. 4). All Cu-CNP priming treatments significantly enhanced the lycopene contents under all conditions whereas the maximum impact was induced by treatments with 0.12% solution (Table 4).

**Table 4:** Effect of Cu- CNP priming treatments on pigments

Pigments	Treatments											
	Non- Stress				Under PEG-induced hyperosmotic stress				Under salt stress			
	Control	Hydro-priming	0.12% Cu- CNP	0.16% Cu- CNP	Control	Hydro-priming	0.12% Cu- CNP	0.16% Cu- CNP	Control	Hydro-priming	0.12% Cu- CNP	0.16% Cu- CNP
Chlorophyll a (ug/g F.wt.)	207 de	85 f	300 a	223 c	219 cd	83 f	259 b	298 a	202 e	83 f	297 a	291 a
Chlorophyll b (ug/g F.wt.)	39 d	16 e	53 c	64 ab	33 d	15 e	68 a	58 bc	51 c	17 e	52 c	57 c
Total chlorophyll (ug/g F.wt.)	246 d	101 e	353 a	287 c	251 d	98 e	328 b	356 a	253 d	101 e	350 a	348 a
Β-carotene (mg/g F.wt.)	3.7 d	2.2 g	5.6 a	4.5 c	4.3 c	2.2 g	3.3 e	4.8 b	2.9 f	2.2 g	5.7 a	4.7 b
Total carotenoids (mg/g F.wt.)	15 b	7 c	23 a	20 a	16 b	8 c	23 a	24 a	15 b	6 c	23 a	23 a
Lycopene contents (mg/g F.wt.)	3.04 d	1.34 e	4.7 a	4.05 b	3.15 d	1.31 e	4.7 a	3.8 bc	3.16 d	1.33 e	4.7 a	3.6 c

Mean values of three replicates presented. Within a column, means sharing the same letters are non-significantly different (*p* > 0.05 and *p* > 0.01) according to the Tukey’s Test (HSD)

Except for treatment with 0.12% Cu-CNPs under PEG-induced hyperosmotic stress, all other Cu-CNP priming induced a significant increase in β-carotenoids under stress and non-stress conditions. Priming treatments under all conditions also caused a significant increase in total carotenoids. Further, they also induced a significant increment in chlorophyll a, b and total chlorophyll under stress and non-stress conditions.

## Discussions

Both drought and salinity cause severe negative effects on seed germination which is the most critical stage and directly controls the growth and subsequent yield of crops. Salinity disturbs the germination parameters due to the specific toxicity of ions which inhibit the cell division and expansion processes. It also reduces the water uptake in germinating seeds [4, 42]. In our study, priming with Cu-CNPs helped to reduce the negative impacts of both stresses on germination rate. There was no significant difference in germination percentage and mean germination percentage under control and stress conditions which suggests the stress mitigating role of Cu-CNP priming treatments. Behboudi et al. used foliar and soil applications of chitosan nanoparticles to mitigate the hazardous effects of drought on barley with the improvement of yield and yield components [43]. According to Choudhary et al. the Cu-CNP seed treatments enhanced the shoot length and vigor index but did not affect the germination percentage of maize seedlings under normal conditions [44]. The foliar applications of Cu-CNPs also exhibited the growth promotory effect in terms of root length, plant height and stem diameter etc. in maize plants in pot and field experiments [26]. In our study, the enhancement of root length under salt stress while no significant change under PEG-induced hyperosmotic stress represents the mitigating role of Cu-CNP priming against both stress conditions. Similarly, both stresses were unable to produce any significant effect on shoot length, again establishing the stress insulating effect of Cu-CNP priming.

The plant productivity is determined mainly by seed germination, a vital stage in plant development. It begins with the water imbibition, protein synthesis and mobilization of food reserves. The carbohydrates, lipids and proteins are the main food reserves mainly required for phase II of germination leading to better seedling development. Proteins are a vital source of carbon, nitrogen, amino acids and energy for seedling development [45]. Various enzymes being protein in nature execute many vital biochemical processes like respiration, regulation of metabolic pathways, biosynthesis of macromolecules and development of subcellular structures in seed germinating and seedling development. However, the enzymatic hydrolysis of lipid, carbohydrates and proteins and further transportation of their metabolites heavily depends on the availability of water [46]. It is reported that drought stress-induced proteins help the wheat seedlings in structural and biochemical readjustments as a mechanistic approach to elicit stress tolerance [47, 48]. In our study, the priming treatments induced a significant increase in protein contents in wheat seedlings under PEG-induced hyperosmotic stress which was a positive response in light of the above discussion. Similarly, proteins also play important role in salt stress acclimation in plants. Salinity causes changes in protein activity, protein post-translational modifications and protein relative abundance as a salt tolerance measure in plants including wheat [49]. Salt stress enhances the protein contents in wheat [50] and so was the observation in our case as non-primed control showed significantly high protein contents under salt stress. However, priming with Cu-CNPs induced a significant reduction in protein contents under salt which suggests the lesser requirement of proteins due to the stress mitigating role of Cu-CNP treatments.

Sugars are vital for innumerable functions like the generation of functional metabolites, biosynthesis of biopolymers, energy production and metabolism in plants. In addition to their nutritious value, they act as regulators of stress responses, growth and seedling development [51]. In general, they ensure membrane stability by acting as osmoprotectants under abiotic stress conditions. They scavenge free radicals and provide membrane protection under drought as well as salt stress conditions [52, 53]. However, higher concentrations of sugars could reverse the afore-said physiological processes in a concentration-dependent manner [54]. In our study, no significant change in total sugar contents suggests their fewer requirements as stress insulators as the same role may have been played by Cu-CNPs.

In general, salinity reduces the activity of α-amylase thus influence the starch hydrolysis and liberation of soluble sugars [55, 56]. In our case, both stress conditions reduced the α-amylase activity in seedling originated from Cu-CNP primed seeds while the more pronounced effect was observed under PEG-induced hyperosmotic stress. Proteases are the key regulators of several physiological and biochemical processes and ensure the safe accomplishment of N homeostasis, seed germination, seedling development, plant growth and immune responses. Under biotic and abiotic stress conditions, they are involved in nutrient remobilization through the degradation of stored proteins for better seed germination and seedling development in plants including wheat [57, 58]. In our case, priming with a higher concentration of Cu-CNPs produced a significant enhancement in protease. Generally, plants induce an increment in proteases as an adapted mechanism to cope abiotic stresses like drought and salinity [59].

Both salinity and drought cause overproduction of ROS by hampering normal cellular metabolic processes in plants. Plants have evolved counteracting antioxidant metabolism including non-enzymatic compounds and antioxidant enzymes to detoxify the excessive ROS for the maintenance of cell homeostasis. Generally, the increased activities of antioxidant enzymes like CAT, POD and SOD etc. are positively correlated with drought and salinity tolerance [60]. The improved antioxidant potential of primed seeds implicates the enhanced stress tolerance in seedlings and plants. However, priming treatments do not always enhance the activity and expression of antioxidant enzymes and in such cases, the AsA-GSH cycle is suggested to be the dominant underlying antioxidant mechanism [61]. In our case, priming treatments enhanced the POD activity significantly under salinity however, a significant reduction was observed in its activity under normal and PEG-induced hyperosmotic stress. The SOD activity also decreased under both stress conditions. Both priming treatments produced opposite effects on CAT activity under PEG-induced hyperosmotic stress as well as salinity. The controlled activities of antioxidant enzymes could be correlated with the above discussion.

The priming treatments also reduced the TPC which are the non-enzymatic antioxidant compounds. The decrease in TPC suggests their fewer requirements due to low production of ROS after Cu-CNP priming. The low and controlled production of ROS has also been justified by low MDA contents. The activities of antioxidant enzymes, contents of non-enzymatic antioxidants and suppression of MDA suggest the insulating potential of Cu-CNP treatments against salinity and PEG-induced hyperosmotic stress.

In general, both PEG-induced hyperosmotic stress and salinity reduce the pigments in seedlings thus inhibit photosynthesis activity which ultimately decreases the growth and yield of crops. However, in this study, the priming treatments significantly enhanced the pigment contents compared to control under both stress conditions. The applications of chitosan NPs have been found to enhance the pigment contents in barley under drought supposedly due to the greater production of amino compounds from chitosan [43]. The priming treatments with pure chitosan also enhanced the chlorophyll pigments in wheat under PEG-induced hyperosmotic stress.

Hence, this nanoparticulate system has been suggested as an effective and safer priming agent to counter dangerous abiotic stresses without compromising soil fertility characteristics. This safety profile is ensured by the sustained and controlled release of Cu, low toxicity of chitosan and little requirements of Cu-CNP.

## Conclusion

In conclusion, controlled expression of enzymatic antioxidants and suppression of MDA revealed the stress mitigating role of Cu-CNP against PEG-induced hyperosmotic stress and salinity. The stress-insulating potential has also been reinforced by the enhanced production of photosynthetic pigments. All these priming-induced biochemical changes produced positive effects on seed germination and seedling growth parameters in wheat under PEG-induced hyperosmotic stress and salt stress. A biopolymer-based nanoparticle system has been suggested as an ecofriendly and safer option to counter afore-said stress conditions without compromising soil fertility and environmental safety.

## Data Availability

All data generated or analyzed during this study are included in this published article.

## Abbreviations

Cu-CNP: Cu-chitosan nanoparticle
MDA: Malondialdehyde
PEG: Polyethylene glycol
POD: Peroxidase
CAT: Catalase
SOD: Superoxide dismutase
ROS: Reactive oxygen species
TPC: Total phenolics
